# Complete Genome Sequence of a Lumpy Skin Disease Virus Strain Isolated from the Skin of a Vaccinated Animal

**DOI:** 10.1128/genomeA.00482-18

**Published:** 2018-05-31

**Authors:** Ivana Lojkić, Ivana Šimić, Nina Krešić, Tomislav Bedeković

**Affiliations:** a Department of Virology, Croatian Veterinary Institute, Zagreb, Croatia

## Abstract

Vaccination of cattle against lumpy skin disease (LSD) can cause adverse reactions. Here, we report the first complete genome sequence of an LSD virus strain isolated from the skin of a vaccinated animal. We confirmed that the sequence of the virus used for vaccination remains stable.

## GENOME ANNOUNCEMENT

In 2015, lumpy skin disease was recorded for the first time in Greece and Cyprus, and since 2016, lumpy skin disease virus (LSDV) has been rapidly spreading to neighboring countries (Bulgaria, Macedonia, Serbia, Kosovo, Montenegro, and Albania) (1).

In Croatia, the preventive vaccination program against lumpy skin disease targeting the entire cattle population was approved by the European Commission (2). Adverse reactions to vaccination, which include the formation of generalized skin nodules accompanied by the detection of vaccine virus in blood, saliva, skin, and milk, were recorded (3).

Here, we report the complete genome sequence of an LSDV strain isolated from the skin of a vaccinated animal showing generalized skin nodules (CRO2016).

Cattle were vaccinated with Lumpyvax (10^4^ 50% tissue culture infective dose [TCID_50_]/ml live, attenuated SIS type virus; MSD Intervet South Africa [Pty] Ltd., Spartan, RSA), according to the manufacturer’s recommendations.

The virus was isolated from a biopsy specimen of a skin nodule sampled away from the vaccine injection site and passaged eight times on Madin-Darby bovine kidney (MDBK) cells, as described before (3). The complete genome was determined by next-generation sequencing (NGS) using Illumina technology.

Total DNA was extracted using the DNeasy blood and tissue kit (Qiagen, Hilden, Germany), according to the manufacturer’s instructions. A sequencing library was constructed using the Nextera XT DNA library preparation kit and sequenced using a MiSeq reagent kit version 2 with 2 × 250-bp paired-end sequencing on a MiSeq benchtop sequencer (Illumina).

For genome assembly, a total of 74,945 reads were mapped to the reference genome (GenBank accession number KX764643) using the Geneious software R10 (Biomatters Ltd., Auckland, New Zealand). After manual inspection of the genome reference mapping, no discrepancies were found. Open reading frames were predicted with the Genome Annotation Transfer Utility (GATU) (4) relative to the LSDV vaccine strain Lumpyvax sequence (GenBank accession number KX764643). The obtained genome sequence (150,448 bp) was 100% identical to that of the vaccine virus (GenBank accession number KX764643).

This confirms that vaccine virus can be isolated from the skin of a vaccinated animal and passaged eight times in MDBK cells, with no effect on sequence composition and thus on the nature of the vaccine virus itself.

### Accession number(s).

The complete genome sequence of the isolate CRO2016 has been deposited in GenBank under accession number MG972412.
